# A case of misleading conclusion: a critical reassessment of methodological and interpretive flaws of a recent meta-analysis on the vector role of cat fleas in feline hemotropic Mycoplasma species

**DOI:** 10.1186/s13071-026-07315-2

**Published:** 2026-08-31

**Authors:** Charlotte O. Moore, Erin Lashnits, Michael Lappin, Edward B. Breitschwerdt

**Affiliations:** 1 North Carolina State University, Raleigh, North Carolina USA; 2 University of California Davis, Davis, California USA; 3 Colorado State University, Fort Collins, Colorado USA

Dear Editors,

We appreciate the recent response to our previously published manuscript in your journal, and in the spirit of scientific inquiry, would like to take this opportunity to respond to the author’s comments and critiques. Firstly, we welcome the author’s deep engagement with this subject and the body of referenced work. We regret any omitted or incorrectly recorded data from the meta-analysis component of our study.

In regard to the mistakes in our data collection for the meta-analysis, we thank this author for their attention to detail and for finding these errors. Indeed, we have revised the supplementary table 1 to include the corrected figures for fleas in the Zarea, Abdullah, and Shaw et al. studies [1–3].

For the study utilizing Jensen primers, we acknowledge the correction that Shaw et al. [3] did utilize washing, and they followed their polymerase chain reaction (PCR) with sequencing in 16 of the 44 DNA extracts from fleas removed from cats, finding a match with *M. haemominutum* over a 193 bp fragment of the 16S rRNA gene. We have included here an updated table 1 reflecting the miscategorization of the Shaw study. However, in our opinion, this correction does not change the conclusion that the Jensen primers could have been amplifying *Spiroplasma* spp. DNA, the prevalence of which was not necessarily reduced by washing. The fact that the Shaw study used washing lends further support to our hypothesis, in that even though the fleas were washed, ~40% of them (44/90) yielded positive results from the Jensen primers.

For the studies using non-Jensen primers, the corrected numbers do not significantly change our interpretation. In the Zarea et al. study [1], it is slightly unclear how the proportion should be calculated, since there were two *C. canis* fleas removed from cats that would not have been included on the basis of our inclusion criteria, but the authors do not specify whether all seven hemotropic mycoplasma-positive fleas were *C. felis*. Conservatively, we can estimate this as 7/91, a prevalence of < 8%. Similarly, for the Abdullah et al. study [2], based on our inclusion criteria, we should have included only the results from the flea pools collected from cats, which included 210 pools. However, according to their results: “The species-specific qPCRs for haemoplasma species found that only 3 flea samples were positive for *M. haemofelis* or *M. haemocanis*, all of which were found in *C. f. felis* fleas, 2 of which were collected from cats, and one had no record of the host species.” Therefore, the numerator for this study should also be revised to include only the two positive pools known to be collected from cats. Using 2/210 (1.0%) instead of the reported 3/467 (0.6%) does not make a statistically meaningful difference in the interpretation of these results. Updating the overall proportion of hemoplasma DNA amplified from *C. felis* using the various species-specific (non-Jensen) primers gives us 14/949 (1.5%), which, in our opinion, still suggests that amplification of hemotropic mycoplasma from fleas when using these species-specific primers via quantitative (q)PCR remains exceedingly rare.

When it comes to the role of pooling, it is true that inconsistent pool sizes within studies complicate meta-analysis. As most studies do not make flea pools of a consistent size and do not report the number of fleas in all positive and negative pools, our meta-analysis only aimed to capture any report of pooling. We urge future researchers to include metadata or analysis of the detection of hemoplasmas in flea pools of varying sizes. The questions remaining on pool size from both the Willi et al. [5] and Abdullah et al. [2] studies, and the different interpretations of different readers, highlight the importance of clear and comprehensive methods reporting, including reporting of pool sizes for these types of studies. Additionally, the impact of pool size on the detection of hemoplasma or other bacteria harbored or transmitted by fleas is unknown, again stressing the importance of focusing future research on determining optimal conditions, including pool size, for appropriate detection of bacterial DNA in fleas via PCR.

Despite referencing the influence of pooling throughout the manuscript, Willi et al. [5] unfortunately do not report the number of fleas per pool. That study reports results of 73 fleas from 17 cats, with pools of 1–5 fleas (per Willi et al. [5] Table 1), and the methods state “Some tick and flea samples were pooled for DNA extraction (Table 1); pools consisted of the arthropods of one species and those collected from one animal.” In the results, the authors state “Three ticks and two fleas collected from animals tested positive with real-time PCR for hemotropic mycoplasmas; all positive samples were extracted from individual arthropods,” while in the discussion the authors offer the following as a potential explanation for a low prevalence estimate of hemoplasmas: “The lower sample prevalence in the present study could be explained by the fact that most fleas were analyzed individually and not in pools.” Therefore, it was not possible to separate fleas by pooled or unpooled for meta-analysis. Similarly, in the Abdullah et al. study [2], while the methods reported that fleas were pooled, at least half of the “pools” as reported by the study were single fleas. No further information was available on pool size otherwise from this study, and the effect of single versus multiple fleas per pool was not investigated further. Regardless, noted differences in interpretation of pooling do not change the conclusion that with the use of species-specific primers, there were very few hemoplasma-infected fleas: two individual fleas in Willi et al. [5] and two pools (likely but not definitively consisting of one flea each) out of more than 200 pools in Abdullah et al. [2].

**Table 1:** Primer set, flea pooling, and flea washing are associated with the detection of hemotropic *Mycoplasma* spp

Primer	Washed	Percentage of infected fleas ± 95% CI (# positive/total)
Myco_Hf_F/Myco_Hf_F.1 and Myco_Hf_R [4]	No	45.7 ± 1.9% (148/324)^a^
Myco_Hf_F/Myco_Hf_F.1 and Myco_Hf_R [4]	Yes	17.1 ± 4.2% (44/258)^b^
Other primers	No	1.5 ± 0.3% (11/734)^c^
Other primers	Yes	1.4 ± 0.1% (3/215)^c^

This table presents the percentage of hemotropic *Mycoplasma* (hMyco)-infected fleas reported by the identified manuscripts on the basis of the PCR primers utilized and whether authors utilized a washing step prior to DNA extraction. Primers are grouped into two categories: those employed by Jensen et al. (2001) and other primers. Superscript letters indicate categories that were significantly different from each other by pairwise comparison of proportions post hoc test

In the experimental component of our study, we retested samples from a previously published study by Assarakorn et al. [6] that reported a high prevalence of hemoplasma DNA in fleas using Jensen primers. We acknowledge that one potential explanation for the significantly lower prevalence in the reanalysis could be due to sample storage, though there is a substantial body of research suggesting that DNA remains stable when stored frozen for years [7]. However, the issues with the pooling methodology are likely not related. The reporting of pooling methodology and results in the original Assarakorn study were, like in many of the referenced publications, somewhat nonspecific, leaving it open to misunderstanding and therefore misreporting in our manuscript [8]. According to the primary investigator of the Assarakorn et al. study (M. Lappin) and a co-author of our manuscript, in the original Assarakorn study, fleas were pooled by cat, with many cats having only one flea per pool, but up to a maximum of five fleas per pool. There is no further information available on the number of fleas per pool or the number of cats with only a single individual flea collected. However, in the original Assarakorn et al. study, there were some cats with more than 5 fleas, and those cats had multiple pools created, but only 1 pool was tested, which explains the numerical discrepancy between the 50 pools (from 50 cats) tested in Assarakorn et al. and the 67 pools (from 50 cats) tested in our manuscript. For our reanalysis, aliquots from those original DNA extracts—all 67—were retested at both Colorado State University using the Jensen primers and North Carolina State University using the Manvell primers. Because all pools from all cats were tested (67 pools from 50 cats), the results can be directly compared between only the 50 original pools. Of the previously untested pools, 7/17 (41%) were positive using the Jensen primers, compared with 18/50 (36%) of the previously tested pools. The results are presented more explicitly here in the table (Table 2).

**Table 2:** Flea pools from Assarakorn et al. [6] study, with results shown by the original result and retesting in Moore et al. [8]

Original Assarakorn et al. [6] result	Moore et al. [8] retesting result			
	Mhf	Spiroplasma	Other bacteria	Negative
Mhf or Mt	0	0	0	2
Mhm	0	13	1	1
Negative	0	2	2	29
Not tested	2	1	4	10

In regard to the grouping of Manvell and Jensen primers, in the Manvell et al. study [9], there were initially a large number of positive PCR results that were recategorized in the final analysis due to sequencing showing that these positives were actually *Spiroplasma* spp., not hemoplasma. In meta-analysis, these primer sets were grouped, as they share a reverse primer and display a single base pair shift in the forward primer that is homologous among feline hemoplasmas and *Spiroplasma* spp. of *C. felis.*

Finally, when it comes to the washing component of our experimental study, we acknowledge that due to unknown hemoplasma presence in the fleas, it is impossible to know the effect of washing. We mentioned this in the Discussion section of our original publication. However, this point could have been emphasized more strenuously. Unfortunately, fleas with known hemoplasma infection were not available to study the role of washing fleas in mycoplasma detection. These important experiments remain to be done.

Overall, the experimental evidence for hemoplasma transmission by fleas is minimal, indicating that more robust experiments are needed. Indeed, the hemoplasma experimental infection numbers cited in our study are small, but not excessively small for such a labor-intensive animal study, considering the difficulty of working with *C. felis* and cats in an experimental setting [10]. The results, while limited, at the time of publication, were the only published experimental transmission study using fleas. Clearly, flea transmission of hemoplasmas remains a subject requiring additional investigation. In that regard, another experimental study has been published [11] since our manuscript was published. That study found less support for flea transmission and more support for direct transmission. Specifically, hemoplasma transmission was observed between flea-infested cats sharing the same enclosure (1/6 infected with *M. haemofelis* and 3/6 with *M. haemominutum*), but no transmission was observed between cats in separate enclosures that only permitted flea movement, but not direct cat contact [11]. In comparison, in that study, *Bartonella clarridgeiae* was transmitted among all of the flea-infested cats in the same enclosure, as well as to two of the eight cats in the adjacent enclosures that allowed for flea movement. This demonstrated a clearer role of *C. felis* in transmission of *B. clarridgieae*, a genus known for predominantly arthropod vector-borne transmission, compared with hemotropic *Mycoplasma* spp.

The role of geography in interpreting the prevalence of hemoplasma infection in cats and fleas is also contentious. From the publications included in our study, when compared with the Switzerland study cited by Safwat [12], Japan [13] and Italy [14] present a clearer range of climate types, from subarctic to humid subtropical and tundra to hot-summer Mediterranean climate, respectively [15]. In the Swiss study by Willi et al. [12], there was increased prevalence of hemoplasma infection in cats from the south and west compared with other parts of the country. While the west of Switzerland is a temperate oceanic climate comparable to the north, the south of Switzerland is a subarctic to tundra climate due to the Alps. The findings by Willi et al. do not therefore associate higher hemoplasma prevalence in cats with areas of increased temperature and humidity, and therefore areas predicted to have more abundant *C. felis*. In addition, the Italy and Japan surveys included flea-borne *Bartonella* spp. surveillance in the same [14] or subsequent related [16] manuscripts, allowing for more effective comparisons between hemoplasma and flea-borne *Bartonella* spp.

We agree wholeheartedly with the author that there are many important factors that may influence infection prevalence and/or detection of hemoplasma DNA in fleas, and hope that future studies will focus not only on the role of washing and pooling, but also on species and strain differences, as well as analytical differences between conventional PCR, qPCR, and other molecular detection methods. Indeed, reports continue to acknowledge the off-target amplification of flea microbiome constituents utilizing 16S rRNA primers with inadequate specificity, highlighting the importance of PCR sequencing and robust Basic Local Alignment Search Tool (BLAST) analysis or alignment [17].

In conclusion, it was not our purpose to disregard the high prevalences of hemoplasma documented using Jensen primers, but to caution that those primers may not be as hemoplasma-specific as originally thought at the time of development and validation. In addition, we acknowledge that fleas may be a competent vector; however, geographic surveys and recent evidence are more supportive of direct transmission of hemotropic *Mycoplasma* species among cats. We very much concur with the conclusion that additional studies are needed to clarify the role of fleas as vectors for these cell-wall-deficient bacteria and elucidate the major route(s) of natural transmission for this important feline pathogen.

## Supplementary Information


Supplementary material 1.

## Data Availability

The data utilized for meta-analysis are available in the updated supplementary data file.
